# TF-CBT Training Augmented with a Self-Care Focus: Understanding Facilitators and Barriers to Treatment Implementation

**DOI:** 10.1007/s10597-023-01130-0

**Published:** 2023-05-05

**Authors:** Julie P. Harrison, Esther Deblinger, Elisabeth Pollio, Beth Cooper, Robert A. Steer

**Affiliations:** 1 CARES Institute, Rowan University School of Osteopathic Medicine, Stratford, NJ USA; 2 Department of Psychiatry, Rowan University School of Osteopathic Medicine, Stratford, NJ USA

**Keywords:** TF-CBT, Dissemination, Secondary traumatic stress, Self-care, Qualitative, Training

## Abstract

Clinicians working with youth exposed to trauma may be at increased risk for experiencing elevated levels of stress and symptoms of secondary traumatic stress, which can negatively impact clinician wellbeing and ultimately contribute to reduced access to quality care for clients. An innovative Trauma-Focused Cognitive Behavioral Therapy (TF-CBT) training incorporating self-care practices (i.e., Practice What You Preach; PWYP) was developed to help facilitate the implementation of TF-CBT and to enhance clinicians’ coping and decrease stress. The primary purpose of this study was to determine whether the PWYP-augmented training met three Objectives: (1) increase clinicians’ feelings of TF-CBT competency; (2) improve clinicians’ coping abilities/reduce clinicians’ stress; and (3) increase clinicians’ insight into the benefits and/or challenges clients may experience in treatment. An exploratory aim was also developed to identify additional facilitators and barriers of TF-CBT implementation. The written reflections of 86 community-based clinicians who participated in the PWYP-augmented TF-CBT training were examined using qualitative methods. The majority of clinicians indicated increased feelings of competency and improved coping abilities and/or stress levels; almost half mentioned increased insight into clients’ experiences. The most frequently mentioned additional facilitators were related to elements of the TF-CBT treatment model. Anxiety/self-doubt was the barrier most frequently mentioned, though all clinicians who mentioned this barrier indicated it lessened or resolved over the course of the training. Incorporating self-care strategies into trainings may serve as a facilitator for TF-CBT implementation by enhancing the competency and well-being of clinicians. The additional insights into barriers and facilitators can be used to further improve the PWYP initiative and future training and implementation efforts.

Efforts to enhance access to evidence-based mental health care for children impacted by trauma are critical for reducing widespread mental health disparities (Whitney & Peterson, 2019). Although Trauma-focused Cognitive Behavioral Therapy (TF-CBT; Cohen et al., 2017; Deblinger et al., 2015) is the most widely disseminated intervention for treating trauma-related symptoms in youth (e.g., Sigel et al., 2013), access barriers to this well-established (Dorsey et al., 2017), evidence-based intervention remain. These barriers disproportionally affect youth and families at higher risk for trauma-exposure, including underserved and ethnic and racial minorities (Alegria et al., 2010; Bornheimer et al., 2018; Roberts et al., 2011). In the United States, the majority of youth mental health services are provided in community-based settings, where professional training and support for implementing evidence-based practices (EBPs) are often limited (e.g., Aarons et al., 2009). Continuing to improve the reach of TF-CBT and sustain it in community mental health settings is thus imperative and will require diversified research methods (e.g., qualitative, mixed-methods, experimental) to gain breadth and depth in understanding the facilitators and barriers associated with successful training, implementation, and sustainability in large-scale initiatives.

Factors at the clinician level can impact EBP implementation outcomes across all stages of implementation from exploration and adoption through sustainment (e.g., Aarons et al., 2011). Elevated levels of stress and burnout are commonly experienced by the mental health workforce (Paris & Hoge, 2010), and risk for developing burnout is higher for clinicians working in community-based settings (Yang & Hayes, 2020). Clinicians working with trauma-exposed clients may also experience symptoms of secondary traumatic stress (STS; Hensel et al., 2015), which parallel symptoms of posttraumatic stress disorder (PTSD), such as intrusive thoughts and avoidance (Bride et al., 2004). High levels of burnout may increase clinicians’ vulnerability to developing STS (Shoji et al., 2015). Both burnout and STS can impact self-reported clinical effectiveness and perceived quality of care (Luther et al., 2017; Salyers et al., 2015) and have been theorized to include reduced capacity for empathy and/or compassion (Bride et al., 2004; Maslach & Jackson, 1981). Burnout and STS may also contribute to increased rates of turnover (e.g., Salloum et al., 2015) and the effects of the COVID-19 pandemic may have exacerbated stressors commonly experienced by the mental health workforce (Holmes et al., 2021; Whitt-Woosley et al., 2022). As community-based clinicians working with trauma-exposed clients may be at higher risk for experiencing these negative effects, it may be particularly important to develop targeted implementation strategies to support and retain TF-CBT clinicians when planning for EBP sustainment. In considering the magnitude of stressors faced by community-based clinicians working with trauma-exposed populations, a focus on increasing clinician self-care appears essential for the well-being of clinicians and their clients, and for improving the sustainability of EBPs in community settings.

To help address the above factors, and enhance the implementation of TF-CBT, Deblinger and colleagues (2020) incorporated self-care practices, referred to as “PRACTICE What You Preach” (PWYP), into TF-CBT trainings. The PWYP-augmentation was informed by literature on STS and burnout, and was designed to be integrated into TF-CBT training programs to help facilitate TF-CBT implementation as well as reduce stress levels and improve the well-being of mental health professionals. The PWYP-augmented TF-CBT training model utilizes gold-standard EBP-training approaches (Beidas et al., 2012; Edmunds et al., 2013) and a modified version of learning collaborative methodology (Ebert et al., 2008). The multi-day introductory and advanced trainings and consultation calls include PWYP exercises and assignments designed to parallel the TF-CBT PRACTICE components—**P**sychoeducation and **P**arenting, **R**elaxation, **A**ffective expression and modulation, **C**ognitive coping, **T**rauma narration and processing, **I**n-vivo mastery, **C**onjoint parent-child sessions, and **E**nhancing safety—to help participants cope with stress and reduce possible STS symptoms. The PWYP augmentation also aims to facilitate the implementation of TF-CBT by providing structure and additional opportunities for participants to build competency applying TF-CBT skills by learning, practicing, and sharing, during trainings and consultation calls, their experiences personally utilizing the TF-CBT skills they teach their clients.

To evaluate the effectiveness of the PWYP-augmented TF-CBT training approach, in a pilot study, Deblinger et al. (2020) used a pre-post design to compare ratings from trainees on a number of constructs including self-reported TF-CBT competency, STS symptoms, coping, and use of self-care (i.e., PWYP) skills. Client outcomes were also measured. This pilot evaluation of the TF-CBT learning collaborative augmented with PWYP demonstrated promising results on standardized measures, including significant increases in trainees’ TF-CBT competency and use of coping skills, and significant decreases in STS, as well as positive client outcomes (i.e., decreased PTSD symptoms and behavioral problems).

The purpose of the present study was to gain a deeper understanding of community-based clinicians’ experiences participating in a self-care augmented TF-CBT training program by using qualitative methods to complement and expand on Deblinger and colleagues’ (2020) quantitative assessment of this innovative TF-CBT training approach, which was designed to improve clinician well-being and help facilitate TF-CBT implementation. Our primary aim was to assess whether the three objectives of the training were met by analyzing clinicians’ responses to open-ended prompts about their training experiences. The objectives of the PWYP-augmented training were to: (1) increase clinicians’ feelings of competency in implementing TF-CBT; (2) improve clinicians’ coping abilities/reduce their stress levels; and (3) increase clinicians’ insight into the benefits and challenges clients may experience when implementing TF-CBT skills. For the primary aim, the a priori confirmatory hypotheses were that the majority of participants would report improvements related to each of the three PWYP-augmented training objectives. The development of these objectives was informed by literature on training outcomes as well as STS and burnout related to their effects on clinician well-being and clinical practice. In addition, to help inform ways to enhance the PWYP initiative and future EBP-training, implementation, and sustainability efforts in community-based settings overall, an exploratory aim was developed to identify additional facilitators as well as barriers of TF-CBT implementation mentioned in clinicians’ written responses.

## Methods

Over the course of approximately nine months, community-based clinicians participated in a self-care-augmented (i.e., PWYP) TF-CBT training program that consisted of a two-and-a-half day introductory training, a two-day advanced training, special topics webinars, and 16 one-hour consultation calls. Consultation calls were conducted in groups of 10 to 12 participants and were provided by the trainers. Starting with the in-person introductory training and continuing through the remaining training activities, participants were encouraged to personally practice the skills they were teaching their TF-CBT clients. More specifically, clinicians were asked to intentionally use the PWYP skills with the aim of making an ongoing behavioral change and to focus on skills that they typically used less frequently to help incorporate the new skills into their routine. For example, participants were encouraged to try a new physical activity, increase the frequency of an already used activity, or try to praise someone they did not typically praise. As mentioned above, the PWYP skills were specifically designed to parallel the PRACTICE components of TF-CBT. For example, to parallel the cognitive coping component, clinicians were asked to complete a thought record to challenge their own unhelpful or inaccurate thoughts and replace them with more helpful, accurate thoughts. Participants were asked to utilize praise and reflective listening in their personal lives as a parallel to the parenting component. To parallel the affective expression and modulation component, participants were encouraged to utilize focused breathing and mindfulness. Opportunities for PWYP skills practice were provided during the trainings and consultation calls. PWYP activities that reinforced the skills were assigned as homework. For a detailed review of the PWYP augmentations, see Deblinger et al. (2020). All study activities were approved by the Rowan University School of Osteopathic Medicine Institutional Review Board (Pro2014000110).

### Participants

Three cohorts of community-based mental health clinicians in New Jersey participated in a training program between May 2018 and October 2019. Prior to participating in any learning collaborative activities, 105 clinicians were e-mailed the research consent form that outlined that everyone who participates in a learning collaborative is required to complete the associated surveys. The research consent form also informed participants that providing consent to use survey responses for research purposes posed no added risk to their privacy, and that declining to provide research consent would not impact the training-related services received. Ninety-eight percent (*N* = 103) provided consent for their survey responses to be used for research purposes. Of these consenting clinicians, 86 (83%) clinicians from 19 agencies submitted written responses to the narrative writing prompts used for the current qualitative analyses. The majority of participants were female (90%) with a mean age of 34.41 (*SD* = 8.21) years. With respect to race, 72% of clinicians identified as Caucasian, 14% identified as African American, 4% identified as Biracial, 4% identified as “Other”, 2% identified as Asian or Pacific Islander, and 5% preferred to not answer. With respect to ethnicity, 80% reported being Non-Hispanic/Non-Latino. The majority of clinicians were licensed (85%) and the primary professional fields reported were: social work (57%), professional counseling (26%), marriage and family therapy (7%), psychology (7%), and “other” (4%). In a select-all style question asking about previous training, 27 (31.4%) of the participants had no TF-CBT training, 15 (17.4%) had informal TF-CBT training (e.g., read the TF-CBT book, received TF-CBT supervision from a supervisor who did not participate in the formal TF-CBT Train-the-Supervisor program), and 44 (51.2%) had received some formal training (e.g., completed the TF-CBT*Web* web-based training, attended a one to two-day workshop). None of the participants had previously participated in a TF-CBT learning collaborative nor TF-CBT consultation calls. Sixty-six (75.6%) clinicians reported they had not implemented TF-CBT in the three months prior to beginning the learning collaborative.

### Data Collection

Shortly before the final consultation call, clinicians were asked to complete a narrative writing exercise about their thoughts and feelings related to their experiences in the PWYP-augmented TF-CBT training program. This writing exercise was the PWYP activity parallel to the trauma narration component of TF-CBT and was designed to provide clinicians an opportunity to experience intentionally writing about their thoughts and feelings. Of note, for ethical reasons, clinicians were deliberately not asked to write about traumatic experiences. The timing of the assignment within the training program and the open-response format of the exercise also allowed clinicians to provide quality improvement feedback about the TF-CBT training program and the PWYP self-care focus. Written responses were submitted through Qualtrics, an online survey tool. Clinicians were asked to submit responses to the following two narrative prompts:

Prompt 1: *Please reflect on and share below your thoughts and feelings about how learning and implementing TF-CBT with your clients impacted you personally and professionally.*

Prompt 2: *Please reflect on and share below your thoughts and feelings about how the focus on PRACTICE What You Preach and using the TF-CBT skills for the purpose of self-care impacted you personally or professionally.*

Clinicians were asked to provide their names on a separate line from the prompt responses. Names were used solely to track which participants completed the exercise. Clinicians were informed that only their responses, not their names, would be shared with the trainers/consultants; however, clinicians were not precluded from including identifiable information within their responses to the prompts.

### Data Analysis

Written responses were de-identified prior to being provided to the authors for codebook development. Separate procedures and qualitative approaches were utilized for the primary aim and the exploratory aim; coding for both aims was conducted using Microsoft Excel. Steps were taken to enhance the methodological integrity of the processes used throughout the design and analysis procedures. Guidelines proposed by the American Psychological Association for reporting qualitative research were followed (Levitt et al., 2018).

### Primary Aim

A deductive, directed qualitative content analysis approach was used for the primary aim, as the objectives of the training were pre-defined and guided the development of the codebook (Hsieh & Shannon, 2005). The primary goal was to analyze clinicians’ written responses to assess whether each of the three training objectives were met. As the three hypotheses of the primary aim were confirmatory in nature, a number of efforts were made to reduce potential bias in the coding process, including the creation of two separate teams: a codebook development team and a coding team. The codebook development team was comprised of the first, second, and third authors. The coding team for the primary aim was comprised of two doctoral-level clinicians and one master’s-level clinician. All coders for the primary aim had been trained in TF-CBT and two of the three coders had prior experience with qualitative coding.

The codebook development team created the initial codebook based on a process involving iterative rounds of coding and discussion of the written responses of a separate cohort of 33 clinicians who consented to allow their responses to be used for research purposes, but were not included in the study sample. The written responses from this separate cohort were designated to be used strictly for the purpose of developing the codebook and for training the coding team. The codebook included detailed guidance and descriptions of the coding process, operational definitions of the three objectives, and representative examples. The unit of analysis for the primary aim coding was each clinician’s full written responses to the two narrative prompts. When coding for each of the three objectives, responses could be assigned either a “Yes,” “No,” or “NM” (“Not Mentioned”) code. The appropriate code for each objective was determined by identifying whether or not that objective was clearly met at some point within a clinician’s narrative responses; a “NM” code was assigned when not enough information was present in a response to definitively assign a code of “Yes” or “No.”

Training for the coding team was provided by the first author, who consulted with the second and third authors as needed. Coding team training for the primary aim included multiple meetings, group and independent coding practice, and discussion of discrepancies to reach consensus. To help limit coding fatigue, coders were encouraged to take frequent breaks and code only a few narratives at a time; coders were also encouraged to utilize memoing to help prevent drift (Kleinheksel et al., 2020). Responses were split equivalently between the three coders and coders were provided with a list of randomly assigned responses to code. Forty-seven (55%) randomly selected clinician responses were double coded. After independent coding was complete, 16 discrepancies (out of 141 double codes) were resolved through consensus. For double-coded responses, coder pair agreement was above 80% for each objective with an overall percent agreement of 89% across all coder pairs and objectives.

### Exploratory Aim

As a complement to the primary aim, to inform ways to enhance the PWYP initiative and future implementation efforts, a combined deductive and inductive qualitative thematic analysis approach was used for the exploratory aim to identify additional facilitators of as well as barriers to TF-CBT implementation (Braun & Clarke, 2006). The deductive components involved the creation of a preliminary codebook to guide analysis, which was influenced by a review of relevant literature and an initial scan of clinicians’ responses to the narrative prompts. The inductive components allowed for category development to be generated directly from the narratives and for unexpected themes to emerge during the coding process. Because this aim was exploratory in nature and involved looking at facilitators outside of PWYP as well as barriers to TF-CBT implementation, it was assumed there was less risk of bias affecting coding. Thus, the first three study authors served as the coding team for the exploratory aim.

In the codebook, an additional facilitator was defined as a factor that promotes the implementation of or adherence to TF-CBT; additional facilitators represented factors beyond the facilitators already captured in the primary aim. A barrier was defined as a factor that impedes or hinders the implementation of or adherence to TF-CBT. To gain additional insight into the nature of the reported barriers, a barrier was also given an identifier of “initial challenge” if there was an indication within a clinician’s written response that the barrier had resolved or at least lessened during the course of the learning collaborative.

Before coding, the first author engaged in a data reduction process to identify additional facilitators as well as barriers in the narratives and directly summarized the relevant text into more manageable units to code. Caution was employed to ensure that no interpretation or alteration of meaning occurred (Graneheim & Lundman, 2004). The first three authors reviewed the summarized data independently and, through discussion and initial group coding, inductively derived categories and themes directly from the data. Coding was then completed independently. Discrepancies were resolved through consensus.

## Results

### Primary Aim

**Fig. 1:**
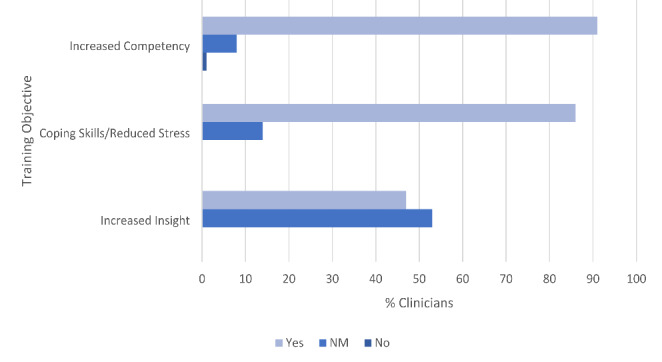
Primary aim: training objectives

Two of the three hypotheses were strongly supported, as shown in Fig. 1. The majority of clinicians indicated improvements related to objective 1 (91%; increase TF-CBT competency) and objective 2 (86%; improve clinicians’ coping skills and/or stress levels). Objective 3 (increase clinicians’ insight into clients’experiences in TF-CBT) was indicated in 47% of the written responses. Thus, the hypothesis for objective 3 was not supported by a majority, although almost half the sample did indicate some increased level of insight. Figure 1 also shows that across all objectives “NM” (“Not Mentioned”) was the second most commonly assigned code (coded 65 times total across all three objectives), and only one “No” code was assigned. Of note, the included quotes are unedited and presented as originally written by participants; limited exceptions to this were made when the quote’s original wording would have caused confusion and detracted from understanding or if the quote contained the name of the trainers/consultants (all edits are indicated with “[ ]”). Additionally, some quotes were shortened to enhance or preserve meaning (as indicated by “…”).

#### PWYP Objective 1: Increased TF-CBT Competency

A large majority of participating clinicians (91%; *n* = 78) indicated an increase in their TF-CBT knowledge and feelings of competency. Clinicians described an increased comfort or confidence in implementing TF-CBT overall as well as in using specific TF-CBT skills/components, particularly the trauma narrative component. Clinicians also often wrote that they felt more knowledgeable about trauma in general and more effective working with trauma-exposed clients. For example, one clinician wrote, “*Learning about the steps involved in TF-CBT has been beneficial… It has helped me to feel more competent in my ability to help a client with traumatic issues.*” Additionally, many clinicians reported a direct link between their increased feelings of competency with their personal use of PWYP skills. For example, one clinician shared, “*I feel that it is important to utilize the skills you teach your clients because it does build your confidence in implementing the treatment model if you become your own expert.”*

#### PWYP Objective 2: Increased Coping Skills and/or Stress Reduction

The majority of clinicians (86%; *n* = 74) indicated improvements in their coping and/or reductions in stress (see Fig. 1). Clinicians often reported the PWYP focus helped them discover new skills they found beneficial or helped them to use skills more frequently. Clinicians also wrote about specific coping skills they practiced and noted a connection between the use of coping skills with an increase in well-being or a reduction of stress. One clinician shared, “*Personally, utilizing the Practice What You Preach exercises has positively changed my interactions with those around me and has served to lessen my own symptoms of stress and anxiety. It has been an important reminder to practice self care, remain mindful and identify when I am having dysfunctional and/or unhelpful thoughts and replace them with more helpful ones*.” Some clinicians’ responses not only noted the benefits of PWYP in terms of managing stress specifically associated with trauma-related work, but also acknowledged its benefits more broadly. For example, one clinician wrote, “*Implementing self-care and Practice what you preach has helped me not only manage my own thoughts and feelings regarding clients with trauma, but my caseload in general.”* Many clinicians also reported noticing the positive impact of their use of skills both personally and professionally. As an example, one clinician wrote, “*The importance of self-care is paramount in a profession with a high exit rate. The word self-care is so often heard it is often glossed over. I often have thoughts of “I do not want to go to work today” or “I hope I have a no show today so I can have a break”. I have guilt over this thought. When I have that thought too often I know I need to reflect and have gratitude over this special position I have in my client’s lives. Having gratitude is also an important lesson in ones personal lives. Practicing mindfulness with loved ones allow for the relationship to prosper and grow which ultimately helps the therapist. Overall, the importance of consistent self-monitoring and self-care cannot be overstated. It allows us to be better therapists and better people*.”

#### PWYP Objective 3: Increased Insight into Clients’ Experiences in TF-CBT

Although the narrative prompts did not specifically instruct clinicians to think about how participation in the training affected their empathy for clients, analyses revealed that 47% (*n* = 40) of the participant sample indicated an increase in their level of insight or understanding of the benefits and/or challenges clients may experience when adopting new TF-CBT skills. To be coded as “Yes,” the written narratives had to indicate that the increased insight into clients’ experiences was directly related to the clinician’s personal experience with PWYP. Clinicians who described this link often noted how their personal use of PWYP skills increased their comfort using the skills and/or increased their belief in their usefulness, which improved their motivation and/or ability to help clients learn and practice the skills. For example, as shared by one clinician, “*Perhaps most important, the focus on self-care has renewed my commitment to preserving my own physical and emotional health by incorporating small [but] significant changes in my daily life. Making the effort to “practice what I preach” has also enabled me to make suggestions based on personal experience when working with clients and parents that are often struggling or overwhelmed*.” Clinicians also often described encountering barriers when they attempted to use PWYP skills themselves and reported these experiences then helped them gain insight into barriers their clients may face when attempting to practice new skills in treatment. For example, one clinician wrote, *“[Engaging in PWYP] has provided insight into how difficult it can be to do these things, which has allowed me to be more patient with my clients. It has made me more compassionate and understanding and it has also shown me just how important it is to help clients utilize as many of these techniques as possible in ways that are reasonable for them to implement.”*

### Exploratory Aim

#### Additional Facilitators of TF-CBT Implementation

**Fig. 2:**
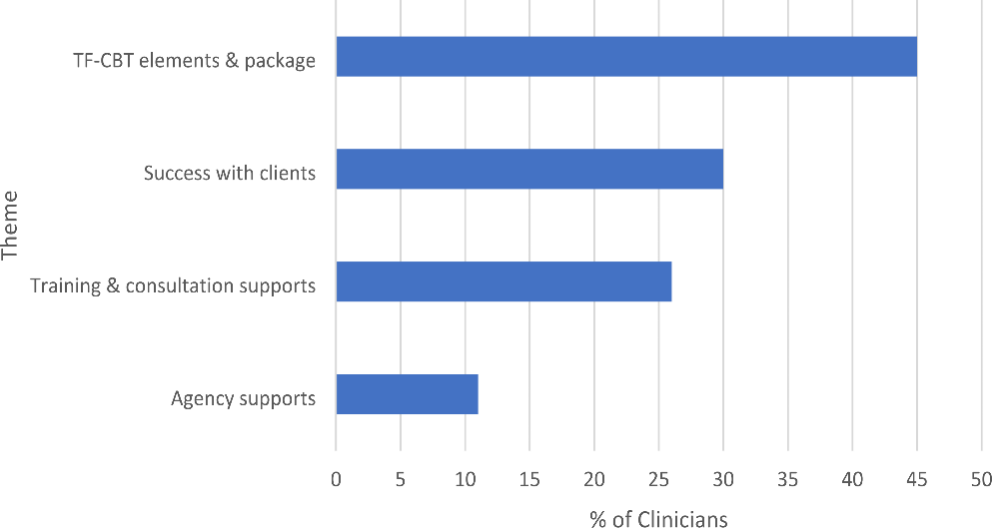
Additional facilitators of TF-CBT implementation

Out of the 86 participating clinicians, 56 (65%) clinicians across 18 agencies mentioned at least one facilitator outside those intentionally built into the PWYP augmentation. Each additional facilitator mentioned in a clinician’s narrative response was coded under one of four mutually exclusive themes: (1) TF-CBT elements and package; (2) Success with clients; (3) Training and consultation supports; and (4) Agency supports. Figure 2 displays the percentages of code counts for each of the four facilitator themes that were identified.

The TF-CBT elements and package-related facilitators were mentioned in 39 (45%) clinician narrative responses. Categories within this theme included receiving and having access to TF-CBT resources and materials; the structure, framework, and focus of the treatment model; the flexibility of TF-CBT and that it is easy to understand; the EBP-status and research support for the model; and the use of assessment measures as part of TF-CBT. Several clinicians noted that the structure/framework provided a guide through the model that supported the learning process. For example, one clinician wrote, “*As for implementing TF-CBT, I have found that having a structured approach actually helped me to feel more organized and confident when planning for counseling sessions. It has also made me feel more effective in my work since I know the success rate of TF-CBT is high.”* A number of responses mentioned the flexibility of the model and/or that clinicians were able to implement the model within their own style.

Seeing client success in treatment, a theme mentioned in 26 (30%) clinician responses, included categories such as observing client and/or caregiver responses to specific skills or to the treatment as a whole, completing a trauma narrative with a client, and completing one or more TF-CBT cases. Success with the trauma narrative, in particular, was noted as impactful for clinicians both within their TF-CBT work, as well as their professional lives more broadly. This sentiment was captured in the following quote: “*The two trauma narratives my clients produced hit an emotional cord within me when they were able to verbalize all that they have learned and gained from coming to therapy. It was a refreshing feeling and reminded me why I went into this field in the first place -- to help people heal.”*

TF-CBT training and consultation support, a facilitator theme mentioned by 22 (26%) clinicians, included categories such as the benefits of learning from expert trainers/consultants, consultant accessibility, and learning from and relating to other trainees on the consultation calls. Clinicians broadly mentioned the availability of the training staff and consultants as being helpful. Clinicians also sometimes mentioned benefits of the consultation calls for specific aspects of their experiences in the collaborative, for example: “*Consultation with [consultant] helped to bolster my confidence in moving forward with the narrative.*”

The agency supports theme, mentioned by 9 (11%) clinicians, included categories encompassing support from TF-CBT supervisors at their agency, having TF-CBT-trained co-workers at their agency, and having agency-based formal or informal TF-CBT groups. Related to agency supports, clinicians highlighted benefits associated with having other TF-CBT-trained clinicians available at their organizations with whom they could consult. For example, as captured in this quote: “*At times, I find myself thinking and seeking counsel in more experienced coworkers and I am grateful for it.”* Additionally, although agency supports were mentioned separately by some clinicians, they were also often paired with discussion of training and consultation support as well (another facilitator theme), with each being noted as important elements that contributed to the overall support they felt they had in implementing TF-CBT. For example, both themes were captured in the following quote: “*When it came to my cases I [definitely] had times [where] I thought “I have no idea what I am doing”. Where I was then able to look back at our training material, ask co-workers, and use the cohort calls to reassure myself and to remind me of the techniques that I knew and needed to be using in the treatment.”*

#### Initial Challenges and Barriers of TF-CBT Implementation

**Fig. 3:**
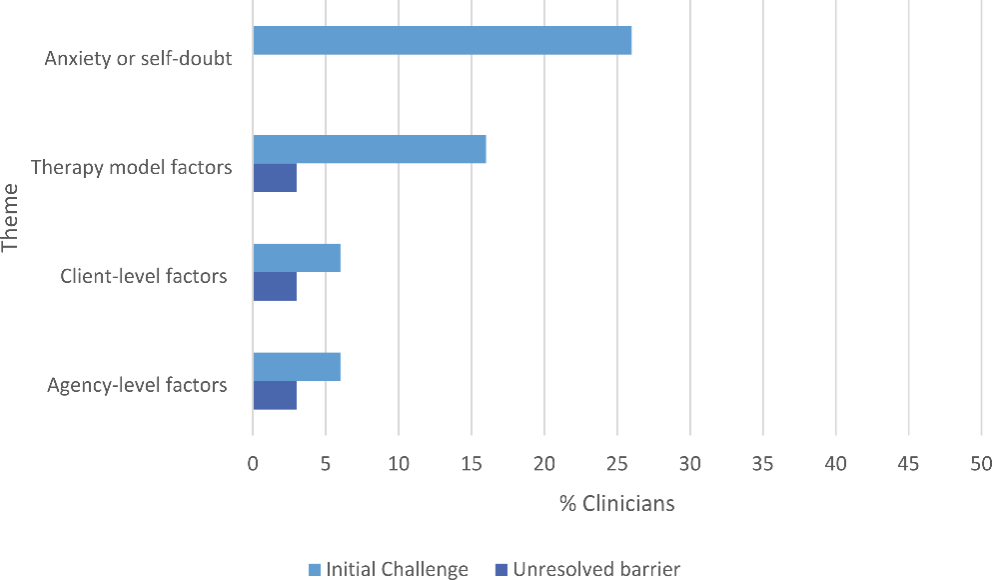
Initial challenges and unresolved barriers to TF-CBT implementation

At least one initial challenge (i.e., a barrier that was indicated to have lessened or resolved over the course of the training program) was mentioned by 34 (40%) clinicians (across 15 agencies) in their narrative response. An initial challenge was coded as one of four mutually exclusive themes: (1) Anxiety or self-doubt; (2) Therapy model factors; (3) Client-level factors; and (4) Agency-level factors. Figure 3 shows the percentages of the code counts for each theme.

Anxiety or self-doubt was an initial challenge mentioned in 22 (26%) clinician responses. This theme included categories that captured statements of self-doubt related to worries about not being good at TF-CBT or good enough as a clinician generally, as well as worries about harming or re-traumatizing their clients. For example, one clinician wrote, “*Before TFCBT I was resistant to address trauma with clients. Because I know how important trauma work is I was afraid I was going to “mess it up”. I was uncomfortable talking about certain traumas with clients. I needed TFCBT training. Because of this training I am able to directly, calmly, confidently, and assuredly talk about trauma with clients…I can let my clients know that they can talk about the most terrible things, and I will be ok, and that they will be ok.*” Another clinician illustrated, in their response, an example of how the number of thoughts related to self-doubt can be overwhelming initially for new learners of TF-CBT, *“Starting this journey was scary. I didn’t know what to expect. Was I going to succeed? Was I going to have what it takes to complete it?…Trying to use TF-CBT for the first time was really scary. Did I explain it correctly? Did I use the right words? Somehow it seemed so much better when the instructors explained it. I had trouble with my first case and felt a little discouraged. Maybe I wasn’t going to be able to do this. Maybe this was too hard for me…Then one day I was in a session and a youth I was working with was really down about his life. He had needed TF-CBT but hadn’t been willing to participate in it before. During our session he finally agreed. I felt like I was really getting [through] to him. The first few sessions went well. We were making really good progress. He seemed as if it was helping him and utilizing some of the strategies he was learning. I felt like I was explaining this right and that I was successful. I had a feeling of accomplishment.“*

Therapy model factors, indicated as an initial challenge by 14 (16%) clinicians, included categories such as limited flexibility of the model, perceived conflicts with therapeutic style, a dislike of manualized treatments, and the time-consuming nature of the model. For example, one clinician noted, *“I’m not a fan of manualized treatments. Not because I don’t respect the evidence based approach but because it feels too restrictive to my therapy style. I was pleased that in the actual work and consultation calls I found ways to make the treatment work with my more free-flowing style.”* Another clinician mentioned how their anxiety and self-doubt (another initial challenge theme) contributed to how time-intensive the model felt initially to implement, *“Initially, there was a lot of anxiety revolving around being effective and staying faithful to the model. I would spend 2–3 hours prepping for one session, re-reading the corresponding chapter in the treatment book over and over. The first couple of months was difficult. I think once i was able to go through each component with clients, my confidence increased. I found that as i began to express my insecurities with my team and co-workers, I was able to challenge many of the anxious thoughts i was feeling using the skills for the model.”*

Client-level factors, a theme indicated by 5 (6%) clinicians, included categories related to cases not completing treatment (e.g., drop out, attendance barriers), encountering client avoidance, and challenges with working with specific client presentations (e.g., young children; comorbidities). Clinicians shared how some of the initial challenges were due to factors outside both their and their clients’ control, as noted in this quote: *“I had some challenges with my clients during this collaborative due to various factors that I knew was not in my control (i.e. insurance lapses), and needed to apply cognitive reframing skills in recognizing this.”* As described in the following quote, some clinicians indicated that certain client presentations initially seemed to present challenges when attempting to apply TF-CBT: *“In the beginning, I was not completely “sold” on the effectiveness of this modality. This was probably because I first used TF-CBT with some of my most challenging cases. I struggled to understand how to apply the components to my work with very young children;…However, with a great support staff helping me navigate through those challenges, and learning how to select developmentally appropriate interventions…I have come to trust the model as a sound and effective model of treatment.”*

Agency-level factors, a theme described by 5 (6%) clinicians, included categories that encompassed difficulties with appropriate case referrals, implementing TF-CBT in non-outpatient settings, inconsistent access to support at their agency, and a busy work schedule. Getting appropriate referrals from their agency for TF-CBT cases was an initial challenge noted by a number of clinicians, as illustrated by one clinician in their response: *“In the beginning of the consultation calls, I did not have any cases for a few months. I had thoughts that I would not get or be able to finish a case in time to complete the collaborative and finish the training…Once I was assigned two cases to work with [and] as I continued in the training and the consultation calls, I felt more sure in my abilities and saw the benefits of using the TF-CBT model with my clients.”* Some clinicians also talked about how they initially thought they did not have or were not given adequate time in their schedules to commit to learning and implementing TF-CBT, given the demands of their job at their agency.

Aside from the above initial challenges or barriers that were overcome or lessened over time, there was a small percentage of clinicians who reported unresolved barriers in their written responses. An unresolved barrier, (i.e., a barrier without any indication that it had lessened or resolved over the course of the training program), was mentioned in less than 10% of narrative responses; more specifically, eight (9%) clinicians (from 7 agencies) noted at least one unresolved barrier. The same four mutually exclusive themes used for coding the initial challenges were utilized for coding unresolved barriers (see Fig. 3). The theme of anxiety or self-doubt was not mentioned as an unresolved barrier by any of the clinicians. The other three themes were each mentioned in 3 (3%) clinicians’ narratives. One clinician noted an unresolved barrier when they spoke to the added load they felt trying to complete the collaborative while managing work expectations of their agency, “*…there were times I struggled to manage my job expectations as well as this collaborative.”* Another clinician noted difficulty associated with trying to implement TF-CBT in non-outpatient settings, “*…I struggled with complications of using the model in a residential setting.”*

## Discussion

PWYP was designed to serve as a facilitator of TF-CBT implementation and improve clinician well-being, with the ultimate goal of finding methods to help expand access to quality mental health treatments for youth. This study aimed to confirm, by analyzing clinicians’ narrative prompt responses, that the three main objectives of the PWYP-augmented TF-CBT training approach were met: (1) enhancing clinicians’ feelings of TF-CBT competency; (2) improving clinicians’ coping while reducing their stress levels; and (3) increasing clinicians’ understanding of the benefits and challenges clients may experience when utilizing TF-CBT skills. As anticipated, it appears that participating in the PWYP-augmented TF-CBT learning collaborative contributed to clinicians’ increased feelings of competence in implementing TF-CBT, enhanced coping, and reduced stress levels, as expressed in their own words. In addition, almost half of the clinicians described increased insight into clients’ experiences when using or attempting to use TF-CBT skills or engaging in TF-CBT components. These findings replicated similar results documented in a quantitative study examining the impact of TF-CBT training augmented with a PWYP self-care focus (Deblinger et al., 2020). However, the qualitative nature of the results of the present study offer reflections, insights, and depth of understanding beyond what could be captured by numerical data. The narrative prompt responses offer a glimpse of the impact of training in much the same way trauma narratives reflect children’s recoveries. Examining clinicians’ narrative responses allowed us to explore the ability of PWYP to serve as a facilitator of TF-CBT as well as identify additional facilitators of and barriers to TF-CBT implementation. The two most frequently mentioned additional facilitators noted by clinicians in their responses were related to the TF-CBT elements and package and experiencing success with clients. A number of initial challenges were shared as well, the two most frequently mentioned of which were anxiety and/or self-doubt as well as therapy model factors. Unresolved barriers were mentioned infrequently. These insights into facilitators and barriers can help inform ways to enhance the PWYP initiative and future training and implementation efforts.

Enhancing feelings of competency is the main objective of any TF-CBT training. The present study documented that over 90% of clinicians described increased feelings of TF-CBT competency in their written responses following their participation in the PWYP-augmented TF-CBT learning collaborative. Learning new evidence-based therapy practices can be intimidating and not something that typically can be successfully accomplished and sustained from a one-time workshop (e.g., Ebert et al., 2008; Lyon et al., 2011). Thus, replicating earlier quantitative results of significantly enhanced competency (Deblinger et al., 2020), the current findings further confirm, in clinicians’ own words, the value of TF-CBT learning collaborative training formats that incorporate multiple learning sessions, consultation calls, and data collection. Additionally, based on clinicians’ written responses, the addition of self-care strategies, as part of the PWYP augmentation, did not appear to detract from the trainees’ learning and implementation of TF-CBT, and potentially may have served to enhance competency by not only encouraging clinicians to practice the skills themselves but by also providing them with opportunities to do so. This endorsement from clinicians may provide some additional support for the acceptability of the PWYP augmentation and provide support for PWYP serving as a facilitator of TF-CBT implementation.

Over 85% of clinicians described improved coping and/or reduced stress as a result of their participation in the training programs. The clinicians’ written responses emphasized the importance of self-care, as part of the PWYP augmentation, in their efforts to manage the stress associated with the field of childhood trauma. As noted directly by some clinicians in their responses, self-care is of great importance because it allows clinicians to not only be better at their jobs but also may offer considerable benefit to them in their personal lives. Though there is some evidence that training in evidence-based practices alone reduces clinician stress (Aminihajibashi et al., 2022), clinicians participating in this learning collaborative specifically cited their use of the self-care PWYP skills as importantly contributing to their reduced feelings of stress and improving their overall well-being. Reducing stress levels among mental health clinicians is also important for improving accessibility to evidence-based treatments given that burnout and STS are common among clinicians working with trauma-exposed youth (Hensel et al., 2015), and may contribute to elevated rates of staff turnover (e.g., Salloum et al., 2015).

The PWYP-augmented TF-CBT training program also helped many clinicians appreciate the challenges and benefits their clients often experience during treatment. Almost half of the clinicians described in their written responses that the personal utilization of PWYP skills increased their understanding of clients’ experiences using the TF-CBT PRACTICE skills and completing related assignments. Such insights, gained from personal experience implementing skills, may enhance clinicians’ feelings of empathy for their clients’ struggles, while also increasing their ability to inspire clients to overcome obstacles to participating in treatment and ultimately implementing and benefitting from the skills. This increased insight may help directly address the reduced capacity for empathy that is theorized to occur with burnout and STS (Bride et al., 2004; Maslach & Jackson, 1981) and may also help clinicians inspire and motivate their clients to follow through with TF-CBT PRACTICE assignments. As client engagement, especially in community-based populations, is often cited as a barrier to successful treatment completion (Dorsey et al., 2014), this additional insight into barriers and motivation to help clients to problem solve these barriers may help with client retention.

To help inform further refinement and enhancement of the PWYP intervention as well as inform training and implementation efforts more broadly, the exploratory aim of this study sought to identify additional facilitators as well as barriers to TF-CBT implementation. Regarding additional facilitators, the most frequently mentioned additional facilitator was the TF-CBT elements and package (i.e., structure/framework and flexibility of the model and associated resources and research support). Although a few participants expressed perceived concerns about the rigidity of the model initially—and most reported this was later overcome—more often the flexibility of the model, and that clinicians were able to implement the model within their style, were highlighted. Another frequently cited additional facilitator was having success with clients. A number of clinicians indicated they initially struggled due to learning a new model, but observing improvements in their clients helped to build their confidence. Success with the trauma narrative in particular was noted, which is an important finding as this TF-CBT component is typically most unfamiliar to clinicians new to TF-CBT and often raises the most anxiety due to hesitancy about how discussing the trauma will impact the family as well as the clinician (Frank et al., 2021). Another additional facilitator mentioned included training and consultation support and availability of consultants. This is consistent with previous research that indicates consultation with experts helps clinicians to not only learn a treatment model but also implement it with fidelity (e.g., Lyon et al., 2011). Although the wording of the narrative prompts likely pulled the least for information about agency-related factors as compared to the other themes, agency support, including co-workers (i.e., supervisors, coworkers, formal or informal TF-CBT groups at the agency), was identified as an additional facilitator by a subset of clinicians. This underscores previous research documenting that agency leadership and support are critical to the successful implementation and sustainability of EBPs (Aarons et al., 2014, 2016).

Initial challenges (i.e., a barrier to TF-CBT implementation indicated to have been lessened or resolved by the end of the training program), were mentioned by almost 40% of clinicians. The most common initial challenge identified was related to anxiety or self-doubt. One quarter of the clinicians wrote about this theme, which highlights how common anxious thoughts and feelings are when first implementing a new evidence-based treatment model. Implications of this finding, as related to training in EBPs, include providing adequate support for clinicians learning a new model, encouraging clinicians to get started with treatment despite this anxiety, and highlighting that this is a common, shared experience so clinicians do not feel alone with any anxiety or self-doubt. Of note, all clinicians who mentioned this challenge also indicated improvements in their comfort implementing the model over the course of the PWYP-augmented learning collaborative training program. In addition to experiencing success with clients, reductions in anxiety and self-doubt may also be partially related to clinicians’ personal utilization of TF-CBT skills (i.e., PWYP). It is also notable that the theme of therapy model factors was more often described as an initial challenge (by 16% of clinicians) versus an unresolved barrier (by 3% of clinicians), as this may indicate that as clinicians proceeded through the learning collaborative, they were often able to observe for themselves the flexibility of the TF-CBT model and figure out how TF-CBT fit with their own therapeutic style. The client-level initial challenges that were mentioned were often related to complex client presentations. The Agency-level initial challenges were often related to delays in obtaining appropriate clients for TF-CBT.

Taken together, the current findings have important implications for the implementation of TF-CBT and can inform trainers, senior leaders, and supervisors of TF-CBT clinicians as well as enhance future PWYP efforts. Related to the PWYP-augmentation, self-care appears to matter. It seems that incorporating self-care into trainings may help to improve clinician competency by increasing opportunities for trainees themselves to practice the skills they will be helping clients to learn. A self-care augmentation to trainings also seems to help with clinician well-being overall which may generally improve the quality of clinical care and potentially result in improved access to TF-CBT in community settings. Additionally, the encouraged practice of self-care skills during trainings may help clinicians with managing anxiety that may come with learning and implementing a new treatment model. Regarding findings related to frequent mentions of anxiety and self-doubt as an initial challenge, and seeing client success as a facilitator, it is also important, as noted above, to acknowledge the normative nature of self-doubt among clinicians implementing a new model while simultaneously encouraging clinicians to get started with clients and work through the model and directly address the trauma despite initial hesitancy. Informing trainees that clients’ narratives have been described by previous trainees as a motivating factor to continue their work in this field may also be helpful. Given the impact of client success, it may also be helpful to highlight during training sessions and consultation calls, as well as agency-provided group supervision, clinical improvements observed throughout treatment. Such improvements to highlight could include symptom reduction as well as clients’ use of skills, progress in trauma narration, and signs of children’s increased resilience (e.g., signs of mastery and improved relationships; Deblinger et al., 2017). The results related to the TF-CBT model suggest the value of outlining, for new TF-CBT clinicians, the structure of the treatment model as a guide while also emphasizing flexibility within that framework. Related to training and consultation and agency supports, findings suggest that providing ongoing support through this process both from the TF-CBT trainers/consultants and peers who are also learning the model is an important component of training as well. In addition, it may be important to identify somewhat less complex cases for trainees when they are implementing TF-CBT for the first time, again, as having initial success seems to build confidence. Although complex cases are highly responsive to TF-CBT (Cohen et al., 2012), new TF-CBT trainees may benefit from gradual exposure to increasingly challenging presentations so they can have early success with clients before moving on to work with clients who present with more complex difficulties and circumstances. In addition, trainers should consult with senior leaders to potentially modify agency screening methods to identify appropriate cases for clinicians early in the training process to increase the likelihood of trainees successfully completing all of the TF-CBT components with their clients while receiving consultation from the trainers.

There are several limitations of the current study. A key limitation is that no follow-up questions could be asked and responses could not be clarified because, prior to coding, written narratives were de-identified. In addition, given the response format and coding methods utilized, the amount of resolution or attenuation that occurred for an “initial challenge” was not measured; therefore, few inferences can be made between initial challenges and barriers. It should also be acknowledged that social desirability bias may have impacted participant responses in a number of ways as clinicians were asked to provide their names for the assignment. Attempts were made to minimize this bias, including informing clinicians that their names would not be shared with their responses, however, it is possible that clinicians still may have felt they needed to reply favorably or positively to the prompts out of concern that their responses could impact the receipt of their certificate of completion for the TF-CBT training program. Additionally, findings may have been impacted due to almost a quarter of participants reporting they had implementeted TF-CBT in some capacity in the three months prior to participating in the learning collaborative. It is possible that barriers and facilitators may have looked different for this subgroup of participants. For example, levels of anxiety may have been lesser for this subgroup when implementing TF-CBT during the learning collaborative since they may have already developed some familiarity with delivering the model. Alternatively, since none of the clinicians who participated in the current study had previously participated in a full TF-CBT learning collaborative or consulation calls, it is also possible that this subgroup could have had greater levels of anxiety when starting the learning collaborative since they had started implementing TF-CBT before having the support from the trainers/consultants. Other limitations include the lack of random assignment or a control or comparison group, which limits the confidence in conclusions that can be drawn.

Future research is recommended in which participants are randomly assigned to TF-CBT training with or without the PWYP self-care focus in order to confirm that the effects of PWYP are not merely a function of the passage of time or an impact of the TF-CBT training alone. Additionally, to tease out the specific effects of PWYP from those of TF-CBT trainings, a clinical trial in which already-trained TF-CBT therapists are randomly assigned to a PWYP self-care course or a comparison condition would be valuable. Finally, although the current study only included clinicians, self-care may be valuable for those in other roles in community mental health agencies as well, and thus, it may be beneficial to include senior leaders, supervisors, and support staff in future research.

In sum, the findings of this study, reflecting the voices of community mental health professionals participating in a TF-CBT training program with a self-care focus, document the reported value of self-care as well as other facilitators and barriers to optimially serving children impacted by trauma. Although the current study’s sample focused on clinicians specifically participating in a TF-CBT learning collaborative, it is likely that mental health clinicians working with trauma-exposed families, regardless of theoretical orientation or preferred therapeutic models, would benefit from training and support that promotes and reinforces self-care practices. Efforts to further develop self-care programs and evidence-based training to support professionals serving in community mental health centers remain critically important and deserving of further research. Given the many challenges faced by mental health professionals, it will also be important to identify alterable factors that might mediate or moderate the impact of such programs on the well-being, productivity, and stability of the mental health workforce. This work would not only positively impact clinicians but ultimately may help improve the availability and accessibility of evidence-based treatments for families in need of such services.
